# In_2_O_3_ Cauliflower Modified with Au Nanoparticles for O_3_ Gas Detection at Room Temperature

**DOI:** 10.3390/nano16010050

**Published:** 2025-12-30

**Authors:** Xiumei Xu, Yi Zhou, Mengmeng Dai, Haijiao Zhang, Jing Xu, Gui Wang, Gang Yang, Yongsheng Zhu

**Affiliations:** College of Physics and Electronic Engineering, Nanyang Normal University, 1638 Wolong Road, Nanyang 473061, China; 20142007@nynu.edu.cn (X.X.); 2024085401005@nynu.edu.cn (Y.Z.); 20252034@nynu.edu.cn (M.D.); 20151007@nynu.edu.cn (H.Z.); xujing@nynu.edu.cn (J.X.); guiwang@nynu.edu.cn (G.W.)

**Keywords:** In_2_O_3_, Au-modified, O_3_ gas sensor, room temperature

## Abstract

Metal oxide semiconductor (MOS)-based chemiresistive gas sensors, attributable to their low cost, compact structure, and long operational lifetime, have been widely employed for the detection and monitoring of trace ozone (O_3_) in environmental air. Moreover, as ozone is a highly reactive oxidizing species extensively used in medical device sterilization, hospital disinfection, and food processing and preservation, accurate monitoring of ozone concentration is also essential in medical sanitation and food safety inspection. However, their practical applications are often limited by insufficient sensitivity and the requirement for elevated operating temperatures. In this study, Au-modified indium oxide (Au-In_2_O_3_) nanocomposite sensing materials were synthesized via a hydrothermal route followed by surface modification. Structural and morphological characterizations confirmed the uniform dispersion of Au nanoparticles on the In_2_O_3_ surface, which is expected to enhance the interaction between the sensor and target gas molecules. The resulting Au-In_2_O_3_ sensor exhibited excellent O_3_ sensing performance under room-temperature conditions. Compared with pristine In_2_O_3_, the Au-In_2_O_3_ sensor with 1.0 wt% Au modification demonstrated a remarkably enhanced response of 1398.4 toward 1 ppm O_3_ at room temperature. Moreover, the corresponding response/recovery times were shortened to 102/358 s for Au-In_2_O_3_. The outstanding O_3_ sensing performance can be attributed to the synergistic effects of Au nanoparticles, including the spillover effect and the formation of a Schottky junction at the Au-In_2_O_3_ interface. These results suggest that Au-modified In_2_O_3_ cauliflower represents a highly promising candidate material for high performance O_3_ sensing at low operating temperatures.

## 1. Introduction

Ozone (O_3_) is a powerful oxidizing agent with important applications in public health and the food industry [1]. Owing to its strong antimicrobial and sterilization capability, ozone has been widely utilized for medical equipment disinfection, hospital environmental hygiene control, and household air purification [2]. In the food processing sector, ozone is widely employed for the inactivation of pathogenic microorganisms, and preservation of food quality owing to its high reactivity and the advantage of leaving no harmful residues [3,4]. However, despite these advantageous applications, uncontrolled exposure to ozone in the environment can pose serious health risks to human and animal health [5]. Even at low concentrations, ozone can cause respiratory irritation, headaches, and ocular discomfort, while long-term exposure increases the likelihood of chronic respiratory diseases [6,7]. At higher concentrations, ozone may lead to severe or even life-threatening effects [8]. Therefore, real time monitoring of ozone concentration and the development of highly sensitive and reliable ozone sensors are of great importance in ensuring safety in related application environments.

Metal oxide semiconductor (MOS) gas sensors are considered ideal candidates for ozone detection owing to their portability, low cost, and rapid response characteristics [9]. In recent years, ozone sensors based on various metal oxides such as In_2_O_3_ [10,11], ZnO [12], CuO [13], SnO_2_ [14], and WO_3_ [15] have been successfully developed, demonstrating reliable sensing performance at elevated operating temperatures. For example, Zhang et al. prepared MOFs derived In_2_O_3_-ZnO hollow microtubes by decorating ZnO nanoparticles onto In_2_O_3_, delivering an enhanced response of 26.12 toward 1 ppm O_3_ at 150 °C [16]. However, in practical applications, such high operating temperatures inevitably lead to increased power consumption and potential safety concerns. Therefore, achieving high ozone sensitivity operating at room temperature remains a significant challenge and a key research focus in the development of next-generation MOS-based ozone sensors.

Research has confirmed that loading noble metals onto metal oxide surfaces can effectively enhance sensor sensitivity and reduce operating temperature [17,18]. Ueda et al. fabricated Au-loaded porous In_2_O_3_ using an ultrasonic spray pyrolysis method with PMMA microspheres as a template. Owing to the Au nanoparticles-induced spillover effect and electronic modulation at the Au-In_2_O_3_ interface, the resulting material exhibited higher concentrations of adsorbed and lattice oxygen, leading to significantly enhanced NO_2_ response and faster recovery under low-temperature conditions [19]. Wang et al. prepared Pd loaded In_2_O_3_ sensors via a reduction precipitation method for room temperature NO_2_ detection, achieving a fivefold enhancement in response compared with pure In_2_O_3_. The Pd induced surface modification effectively regulates the thickness of the electron depletion layer, enabling efficient NO_2_ sensing without the need for additional heating [20]. However, the effect of Au decoration on the O_3_ sensing properties of mesoporous In_2_O_3_ is rarely reported.

In this work, In_2_O_3_ cauliflower was synthesized via a facile hydrothermal route, followed by Au-modification to serve as a catalytic sensitizer. The Au-modification was designed to enhance the sensing response and reduce the operating temperature. The nanostructure and composition of the as-prepared materials were thoroughly characterized, and their gas sensing performances were systematically evaluated in detail.

## 2. Experimental Section

### 2.1. Chemical Materials

Indium nitrate hydrate (In(NO_3_)_3_·xH_2_O, 99.9%), potassium citrate monohydrate (K_3_C_6_H_5_O_7_·H_2_O), sodium citrate dihydrate (Na_3_C_6_H_5_O_7_·2H_2_O), gold chloride trihydrate (HAuCl_4_·3H_2_O, 99.9%), L-lysine were obtained from Shanghai Aladdin Biochemical Technology Co., Ltd., Shanghai, China. In addition, absolute ethanol and deionized (DI) water were used for all of the experiments. All reagents were analytical grade and used as purchased without further purification.

### 2.2. Synthesis of Pristine In_2_O_3_ Cauliflower

In_2_O_3_ cauliflower was synthesized via a simple hydrothermal method. Typically, 0.15 g of K_3_C_6_H_5_O_7_·H_2_O was dissolved in 36 mL of deionized water under vigorous stirred until a clear solution was obtained. Subsequently, 0.3 g of In(NO_3_)_3_·xH_2_O was added, and the mixture was heated to 60 °C and continuously stirred for 1 h. The resulting transparent solution was then transferred into a Teflon lined stainless steel autoclave and maintained at 160 °C for 12 h. After naturally cooling to near room temperature, the precipitate was collected by centrifugation and washed alternately with deionized water and absolute ethanol three times. The obtained product was dried overnight at 60 °C to yield the precursor powder. Finally, the precursor was calcined in air at 550 °C for 2 h in a muffle furnace to obtain the final In_2_O_3_ cauliflower.

### 2.3. Synthesis of Au Modified In_2_O_3_ Cauliflower

Au nanoparticles were deposited onto the In_2_O_3_ cauliflower via a wet-chemical reduction method. In a typical procedure, 50 mg of the as-synthesized In_2_O_3_ cauliflower powder was dispersed in 15 mL of deionized water by ultrasonication for 10 min. Subsequently, 0.31 mL of 0.01 M HAuCl_4_ solution was added dropwise under continuous stirring, followed by the addition of 0.5 mL of 0.1 M L-lysine solution. After stirring for 30 min, 0.5 mL of 0.1 M Na_3_C_6_H_5_O_7_ solution was introduced dropwise, and the mixture was continuously stirred for 12 h. The resulting purple product was collected by centrifugation and washed alternately with deionized water and absolute ethanol three times. The obtained sample was then dried at 60 °C for 12 h and subsequently calcined in air at 300 °C for 30 min in a muffle furnace. After cooling to room temperature, the final product In_2_O_3_ cauliflower modified with 1 wt% Au nanoparticles was obtained and denoted as Au-1.0. To investigate the effect of Au modification on the O_3_ sensing performance, additional samples, containing 0.5 wt%, 1.5 wt%, and 2.0 wt% Au nanoparticles, were prepared under identical conditions and labeled as Au-0.5, Au-1.5, and Au-2.0, respectively.

### 2.4. Material Characterization

XRD patterns were analyzed by X-ray diffractometer (Rigaku Corporation, Tokyo, Japan) equipped with Cu Kα radiation (λ = 1.5406 Å). X-ray photoelectron spectroscopy (XPS) measurements were performed by a Thermo Scientific K-Alpha (Thermo Fisher Scientific, Waltham, MA, USA). The specific surface area and pore distribution of the samples were measured by Micromeritics ASAP 2460 (Micromeritics Instrument Corporation, Norcross, GA, USA). The nanoscale morphology of the sample was observed using a field-emission scanning electron microscope (SEM) model JSM-7800F (JEOL Ltd., Tokyo, Japan) and a transmission electron microscope (TEM) model JEM-F200 (JEOL Ltd., Tokyo, Japan).

### 2.5. Fabrication and Testing of the Sensor

The gas sensors were fabricated using a conventional coating technique. Specifically, 10 mg of the synthesized sample was dispersed in 2 mL of ethanol and thoroughly ground to obtain a uniform slurry. The resulting slurry was then coated onto the outer surface of a commercial ceramic tube equipped with a pair of Pt wires and two Au electrodes. The operating temperature of the sensor was controlled by a Ni-Cr alloy heater located inside the ceramic tube. After coating, the ceramic tube was placed in a muffle furnace and sintered at 200 °C for 2 h to ensure strong adhesion and effective electrical contact between the sensing layer and the ceramic substrate. Subsequently, the sintered ceramic tube and the Ni-Cr alloy heating wire were welded to the sensor base, and the entire device was aged in air at 400 °C for 48 h to further improve the long-term stability and repeatability of the sensor. The sensor response was defined as the resistance ratio, expressed as R_a_/R_g_ in reducing gases and R_g_/R_a_ in oxidizing gases, where R_g_ and R_a_ represent the resistance in the target gas and in air, respectively. The response and recovery times were defined as the time required for the sensor resistance to reach 90% of the total resistance change during the adsorption and desorption processes. Resistance signals were recorded using a UT8806 measurement system (UNI-TREND Technology Co., Ltd., Dongguan, China). The testing procedure and schematic illustration of the sensor configuration are shown in Figure S1.

## 3. Results and Discussion

### 3.1. Structure and Morphology Characterization

**Figure 1:**
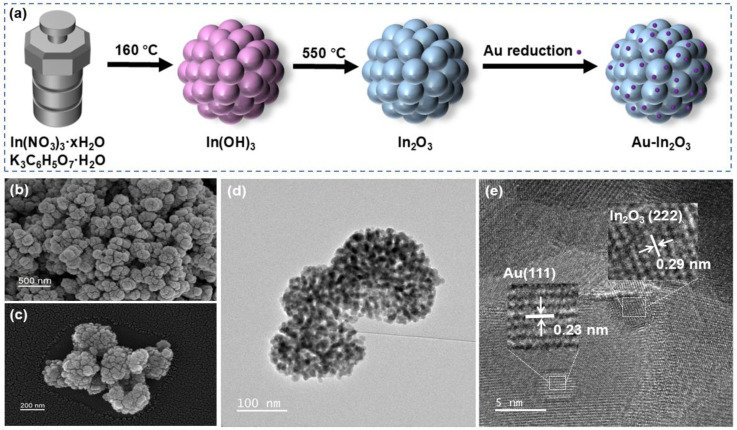
(**a**) Schematic of synthesis of Au-In_2_O_3_ cauliflower. (**b**,**c**) SEM images, (**d**) TEM image, and (**e**) high-resolution TEM (HRTEM) image of the Au-1.0 sample.

Figure 1a presents the schematic illustration of fabrication process of pristine In_2_O_3_ and Au-In_2_O_3_ cauliflower. First, an indium hydroxide precursor was synthesized via a simple hydrothermal method using indium nitrate hydrate as the indium source and potassium citrate monohydrate as the coordinating agent. Subsequently, the indium hydroxide was calcined at 550 °C for 2 h to convert it into indium oxide. Finally, Au nanoparticles were deposited on the surface of In_2_O_3_ cauliflower by chemical reduction in HAuCl_4_·3H_2_O with Na_3_C_6_H_5_O_7_·2H_2_O.

**Figure 2:**
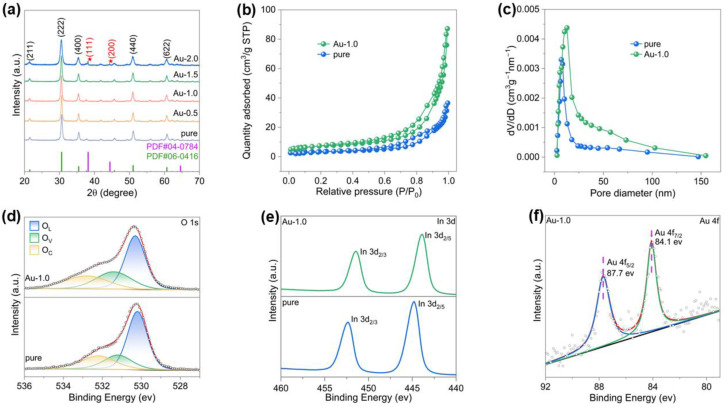
(**a**) XRD patterns of the samples. (**b**) N_2_ adsorption–desorption isotherms and (**c**) BJH pore-size distribution of pure and Au-1.0. XPS spectra of: (**d**) O 1s, (**e**) In 3d_3/2_ and 3d_5/2_, and (**f**) Au 4f for the corresponding samples.

In Figure 1, TEM and SEM images were employed to investigate the structural characteristics and surface morphologies of Au-In_2_O_3_. Figure 1b–d show that the Au-In_2_O_3_ composite exhibits a cauliflower-like hierarchical structure constructed by the aggregation of nanoparticles. The HRTEM image of Au-In_2_O_3_ (Figure 1e) shows lattice spacings of approximately 0.29 and 0.23 nm which are indexed to the (222) plane of cubic In_2_O_3_ and the (111) plane of Au nanoparticles. Powder X-ray diffraction (XRD) analysis was employed to confirm the crystal structure and phase composition of the as obtained samples. As shown in Figure 2a, the XRD patterns of pure, Au-0.5, Au-1.0, Au-1.5, and Au-2.0 exhibit distinct diffraction peaks at 21.4°, 30.6°, 35.5°, 51.0° and 60.6°, which can be indexed to the (221), (222), (400), (440) and (622) crystal planes of cubic In_2_O_3_ (JCPDS no. 06-0416), respectively. No characteristic peaks of Au nanoparticles were observed for the Au-0.5, Au-1.0, and Au-1.5 samples, which may be attributed to the relatively low Au modification. When the Au content increased to 2.0 wt%, two new diffraction peaks appeared at 38.21° and 44.40°, corresponding to the (111) and (200) planes of metallic Au, respectively. All observed diffraction peaks could be indexed to cubic In_2_O_3_ and metallic Au, and no additional peaks attributable to secondary phases were detected, demonstrating that the samples possess high phase purity. Furthermore, the average crystallite sizes of the samples were evaluated from the dominant diffraction peaks using the Debye–Scherrer relationship, expressed as [21]:

(1) $$D=\frac{k\lambda }{\beta cos\theta }$$

(D, grain size; k, Scherrer constant 0.89; λ, X-ray wavelength 0.15406 nm; β, peak width at half-height; *θ*, diffraction angle). Based on this calculation, the mean crystallite sizes of pure In_2_O_3_, Au-0.5, Au-1.0, Au-1.5, and Au-2.0 were determined to be 18.9, 17.7, 16.9, 15.8, and 11.9 nm, respectively, indicating a gradual reduction in crystallite size with increasing Au loading.

The surface properties of the samples were analyzed by N_2_ adsorption tests as shown in Figure 2b, and all samples exhibit type IV isotherms with H3 hysteresis loops, indicative of mesoporous properties. The specific surface areas of the two samples measured by the BET method are 12.95 m^2^/g (pure) and 26.42 m^2^/g (Au-1.0), respectively. High specific surface area supports are crucial for the development of precious metal modified catalysts, as they provide more modification sites, enhance metal dispersion, and improve the utilization efficiency of precious metals [22,23,24]. The deposition of Au nanoparticles not only introduces active sites but also affects the stacking and aggregation of In_2_O_3_ nanocrystals, resulting in more loosely packed structures and thus increased surface area. As shown in Figure 2c, the corresponding BJH pore size distribution curves indicate that after Au nanoparticles were deposited onto the In_2_O_3_ surface, the average pore size increased from 13.9 nm to 18.4 nm, and the pore size distribution broadened accordingly [25,26,27]. The Au nanoparticles partially occupy small pores and induce slight rearrangement of In_2_O_3_ grains during the synthesis and subsequent calcination, leading to larger mesopores and higher pore volume. The abundant pores accelerate gas transport during the sensing process, thereby enhancing sensing performance [28,29,30].

To investigate the surface chemical states and elemental compositions, X-ray photoelectron spectroscopy (XPS) analyses were carried out. As shown in Figure S2, the complete spectrum of Au-1.0 displays that Au, In, C, and O elements exist on its surface. The binding energy values were calibrated using the C 1s peak at 284.5 eV as a reference. As shown in the O 1s spectra (Figure 2d), the O 1s core level peaks of all samples can be deconvoluted into three components in order of increasing binding energy, corresponding to lattice oxygen (O_L_), oxygen vacancies (O_V_), and chemisorbed oxygen (O_C_). The detailed fitting parameters for the pure and Au-1.0 samples are summarized in Table S1. It is worth noting that slight binding energy shifts were observed among different samples, which can be attributed to variations in the local chemical environment. The relative concentration of oxygen vacancies was calculated from the ratio of the integrated area of the O_V_ component to the total O 1s peak area. The oxygen vacancy ratios for the pure and Au-1.0 samples were determined to be 20.8% and 26.6%, respectively. These results clearly indicate that, compared with the pristine cauliflower-like In_2_O_3_, the introduction of Au nanoparticles increases the concentration of oxygen vacancies. In addition, the catalytic activity of Au promotes oxygen activation and spillover processes, thereby stabilizing and exposing a higher density of oxygen-vacancy-related active sites [28,31].

The high resolution XPS spectra of In 3d are presented in Figure 2e, where the two peaks are assigned to In 3d_5/2_ and In 3d_3/2_, respectively. Compared with the pure sample, a slight positive shift in binding energy was observed for the Au-1.0 sample, which could be ascribed to the strong electronic interaction between the noble metal and the metal oxide semiconductor (MOS) matrix. Due to the difference in work functions (Au: 5.1 eV; In_2_O_3_: 2.39 eV), electrons are expected to transfer from the In_2_O_3_ to the Au to achieve Fermi level equilibrium. To further confirm the successful incorporation of metallic Au, the Au 4f XPS spectrum of the Au-1.0 sample was analyzed, as shown in Figure 2f. Two distinct peaks observed at binding energies of 84.1 eV and 87.7 eV can be assigned to Au 4f_7/2_ and Au 4f_5/2_, respectively, confirming the successful modification of Au nanoparticles onto the surface of In_2_O_3_.

### 3.2. Sensing Properties

**Figure 3:**
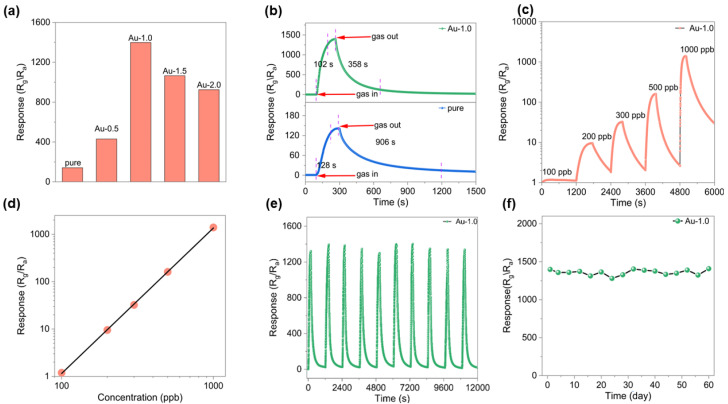
All tests were conducted at 30 °C. (**a**) Responses of sensors based on different samples toward 1 ppm O_3_ gas. (**b**) Response and recovery curves toward 1 ppm O_3_. (**c**) Dynamic responses of the Au-1.0 sensor to different O_3_ concentrations. (**d**) The linear relationship between the sensor response and different O_3_ concentrations. (**e**) Repeatability of the Au-1.0-based sensor toward O_3_. (**f**) Long term stability of the Au-1.0 sensor toward 1 ppm O_3_.

To investigate the effect of Au modification on the gas sensing performance, a series of sensors with different Au contents were fabricated and evaluated toward various concentrations of ozone (O_3_) under room temperature conditions with a relative humidity of 50%. As shown in Figure 3a, the response of sensors with varying Au modification to 1000 ppb O_3_ was compared. The results clearly demonstrate that Au decoration significantly enhances the sensing response of In_2_O_3_. With increasing Au modification from 0.5 wt% to 2.0 wt%, the response initially increases with Au content and then decreases, reaching a maximum at 1.0 wt% The initial enhancement can be attributed to the catalytic activity of Au nanoparticles, which promote the adsorption and activation of oxygen species. However, excessive Au modification leads to aggregation of Au nanoparticles, forming larger clusters, reducing the number of active surface sites and thereby deteriorating the sensing performance [32,33].

The transient response curves of the pure and Au-1.0 sensors toward 1 ppm O_3_ at room temperature are presented in Figure 3b. Both devices exhibit typical n-type semiconductor behavior: the resistance increases upon exposure to O_3_ and returns to its baseline value once O_3_ is removed. Notably, the Au-1.0 sensor exhibits a much higher response (S = 1398.4) compared with that of the pristine In_2_O_3_ sensor (S = 141.5), corresponding to an enhancement factor of approximately 10. Furthermore, the response and recovery times were markedly improved from 128/906 s for pure In_2_O_3_ to 102/358 s for Au-In_2_O_3_. As shown in Figure 3c, the Au-1.0 sensor exhibits responses of 1.2, 9.6, 32.3, 161.2 and 1398.4 to 100, 200, 300, 500, and 1000 ppb O_3_, respectively. Further information can be obtained from the linear dependence between the sensor response and gas concentration (Figure 3d). It should be noted that this curve is presented on logarithmic coordinates, reflecting the sensor’s excellent gas-sensing performance. The actual detection limit is 100 ppb when R_g_/R_a_ ≥ 1.2 is used as the criterion for reliable gas sensing [21]. Stability is a key parameter for evaluating sensor performance. The reproducibility and stability of the Au-1.0 sensor were examined through five consecutive gas cycling tests (air-O_3_-air) at room temperature. As shown in Figure 3e, the response and recovery curves of the ten cycles remain nearly identical under identical testing conditions (1 ppm O_3_ at room temperature). Figure 3f illustrates the variation in the Au-1.0 sensor’s response toward 1 ppm O_3_ over 60 days. The results show that its response remains stable, indicating excellent long-term stability and great potential for continuous monitoring applications. In addition, Figure S3 presents the selectivity tests of the Au-1.0 sample at 30 °C toward 1 ppm O_3_, NO_2_, SO_2_, H_2_S, NH_3_ and 100 ppm of ethanol, acetone, toluene, and CO. The results reveal that the response to 1 ppm O_3_ (~1398) is significantly higher than those to the interfering gases, confirming the outstanding gas selectivity of the Au-1.0-based sensor toward ozone.

### 3.3. Sensing Mechanism

**Figure 4:**
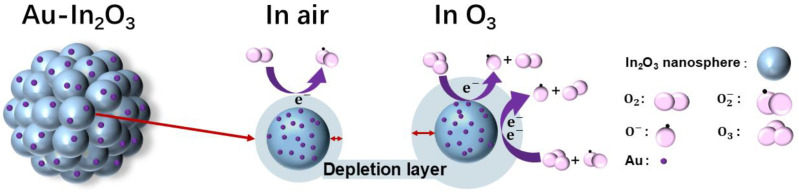
Schematic diagram of the O_3_ sensing mechanism on Au-In_2_O_3_.

Figure 4 schematically illustrates the gas-sensing mechanism of the Au-1.0 sensor. For surface resistance-controlled gas sensors based on n-type metal oxide semiconductors (MOSs), the sensing behavior originates from variations in charge carrier concentration induced by gas adsorption–reaction–desorption processes occurring on the material surface [34]. However, in polycrystalline MOS systems composed of nanosized grains, these carrier concentration changes are not directly translated into bulk conductivity variations. Instead, the electrical response is predominantly governed by the modulation of potential barriers at grain boundaries, which act as the primary transduction elements in such systems [35]. The cauliflower-like In_2_O_3_ architecture consists of numerous interconnected nanocrystalline grains and thus represents a typical polycrystalline n-type semiconductor [36]. At the interfaces between adjacent grains, electron depletion layers formed on individual grain surfaces overlap, giving rise to grain boundary potential barriers. Charge transport through the material therefore proceeds mainly via thermionic emission or tunneling across these grain boundary barriers rather than through the grain interiors. In ambient air, oxygen molecules are chemically adsorbed on the surface of In_2_O_3_ grains and capture electrons from the conduction band, forming ionized oxygen species [37]. Under low-temperature conditions (≤150 °C), molecularly adsorbed O_2_^−^ species dominate on the MOS surface, whereas O^−^ and O^2−^ become prevalent at elevated temperatures. When the sensor is exposed to O_3_, a strong oxidizing gas, additional electrons are extracted from the In_2_O_3_ grains, leading to a pronounced expansion of the surface electron depletion layers. This expansion results in a significant increase in the height and width of the grain boundary potential barriers [38]. Because electron transport in polycrystalline MOS materials is highly sensitive to grain boundary barrier modulation, even small changes in barrier height can induce substantial variations in electrical resistance. Consequently, O_3_ adsorption effectively amplifies the grain boundary barriers, suppresses intergrain electron transport, and manifests as a sharp increase in sensor resistance [39]. The surface reactions occurring at 30 °C can be described as follows [40]:

(2) $$O_{3}+O_{2(ads)}^{-}+2e^{-}\to O_{2}+3O^{-}$$

(3) $$O_{3}+e^{-}\to O_{2}+O^{-}$$

The introduction of Au nanoparticles further strengthens this sensing mechanism governed by grain boundary potential barriers, primarily through the formation of a Schottky contact at the Au-In_2_O_3_ interface and the associated interfacial electron transfer processes. Due to the distinct work function difference between Au and In_2_O_3_, a Schottky barrier is established at the metal-semiconductor interface, introducing an additional potential barrier that couples with the intrinsic grain boundary barriers [41]. Upon contact, electrons transfer from In_2_O_3_ to Au, resulting in electron accumulation on the Au nanoparticles and the formation of a widened depletion layer on the In_2_O_3_ side. This interfacial barrier acts as an effective electron sink, which further modulates the local carrier distribution and enhances the sensitivity of the sensor to surface charge variations. During O_3_ exposure, the Au-In_2_O_3_ Schottky junction facilitates electron transfer from In_2_O_3_ toward Au and surface-adsorbed oxygen species, continuously enlarging the depletion regions at both the metal-semiconductor interface and the grain boundaries. As a result, the grain boundary barriers in the Au-modified In_2_O_3_ become more sensitive to O_3_ adsorption, leading to larger resistance modulation compared with pristine In_2_O_3_ [42]. Due to the spillover process, more oxygen-vacancy-related active sites are exposed. These oxygen vacancies enhance interfacial electron exchange and provide additional reaction sites, thereby further strengthening the coupling between surface reactions and grain boundary barrier modulation. In summary, the superior O_3_ sensing performance of the Au-modified In_2_O_3_ cauliflower structure fundamentally arises from a charge transport mechanism controlled by grain boundary potential barriers, combined with Schottky-contact-induced interfacial electron transfer and barrier amplification effects. The synergistic interaction between intrinsic grain boundary barriers and the Au-In_2_O_3_ Schottky junction enables the efficient transduction of O_3_-induced electron depletion into pronounced resistance changes, thereby realizing highly sensitive O_3_ detection at room temperature.

## 4. Conclusions

In summary, Au-modified In_2_O_3_ cauliflower-like nanostructures were successfully synthesized through a hydrothermal process followed by a chemical reduction method. XRD, SEM, and TEM characterizations confirmed that Au nanoparticles were uniformly dispersed on the surface of the In_2_O_3_ cauliflower. Gas-sensing measurements revealed that the sensor with 1.0 wt% Au modification exhibited the highest response of 1398.4 toward 1 ppm O_3_ at room temperature, which is approximately 10 times higher than that of pristine In_2_O_3_. The enhanced O_3_ sensing performance can be reasonably attributed to the combined effects of charge transport controlled by grain boundary potential barriers and the formation of Au-In_2_O_3_ interfacial Schottky junctions, which together amplify the resistance variation induced by O_3_ adsorption. In addition, the hierarchical porous architecture is beneficial for gas diffusion, while the catalytic spillover effect of Au nanoparticles facilitates oxygen activation and interfacial charge transfer. These results indicate that regulating grain boundary barriers and metal-semiconductor interfacial electronic structures is an effective strategy for developing high-performance room-temperature O_3_ gas sensors.

## Data Availability

The original contributions presented in this study are included in the article/Supplementary Materials. Further inquiries can be directed to the corresponding authors.

## Supplementary Materials

The following supporting information can be downloaded at: https://www.mdpi.com/article/10.3390/nano16010050/s1, Figure S1: Schematic of the sensors and the gas sensing test equipment; Figure S2: XPS spectra of the obtained materials; Figure S3: Responses of the Au-1.0 gas sensor to various kinds of detected gases at 30 °C; Figure S4: Band diagram of the Au-In_2_O_3_; Figure S5: SEM images of the pristine In_2_O_3_ sample; Figure S6: N_2_ adsorption desorption isotherms, pore size distributions and BET surface areas of different samples; Figure S7: Response and recovery curves of toward 1 ppm O_3,_ respectively; Table S1: Peak position and surface oxygen specie contents of the samples; Table S2: The comparison in O_3_ sensing performance of metal oxide-based sensors between the reported studies and our work. References [43,44,45,46,47] are cited in the Supplementary Materials.
