# Supplementary material for: In2O3 Cauliflower Modified with Au Nanoparticles for O3 Gas Detection at Room Temperature
Source: Nanomaterials (Basel). 2025 Dec 30;16(1):50. doi: 10.3390/nano16010050 (PMC12787911; doi:10.3390/nano16010050)
Supplement: Supplementary file 1 [file nanomaterials-16-00050-s001.zip › nanomaterials-4049775-supplementary.pdf]

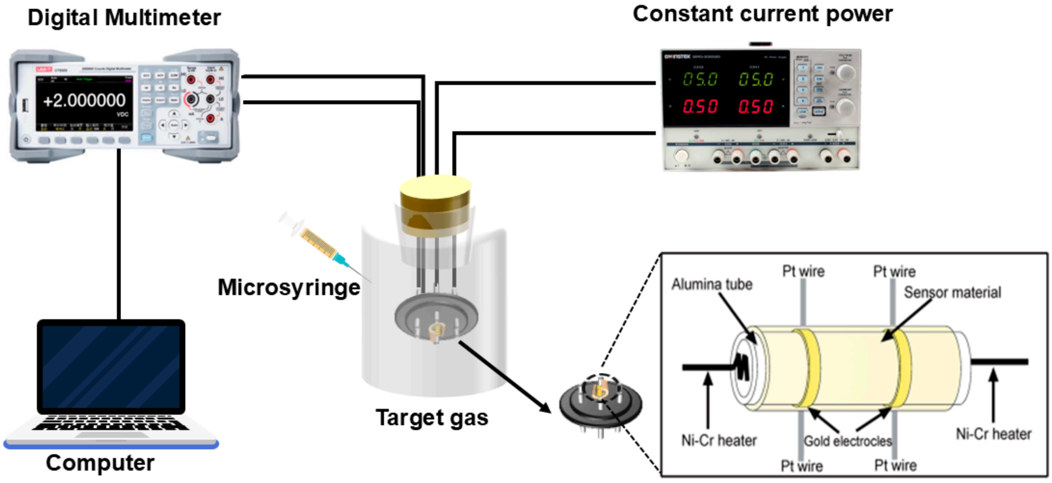

Figure S1. Schematic of the sensors and the gas sensing test equipment.

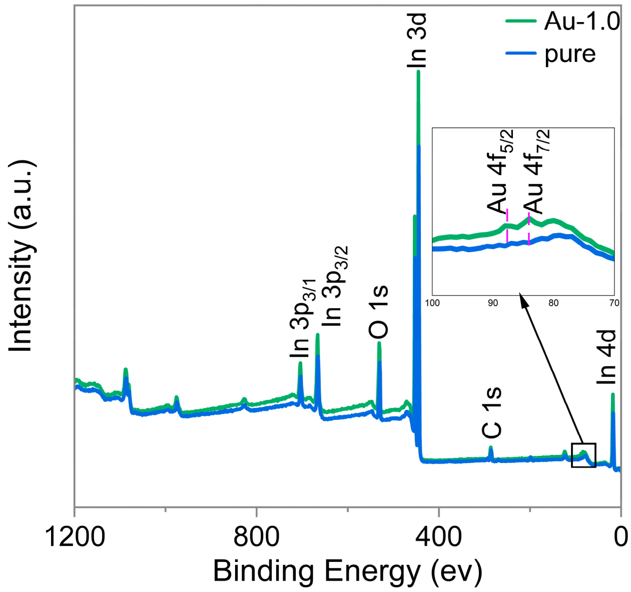

Figure S2. XPS spectra of the obtained materials.

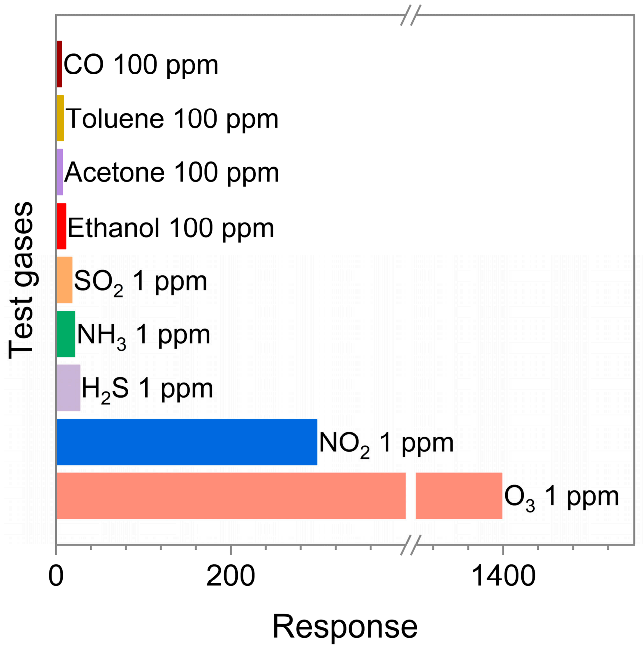

Figure S3. Responses of the Au-1.0 gas sensor to various kinds of detected gases at 30 ºC.

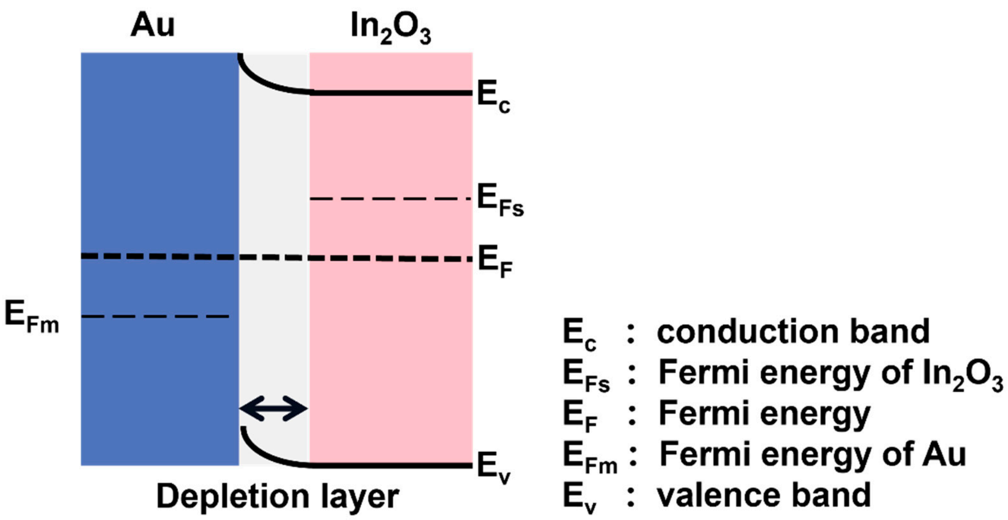

Figure S4. Band diagram of the Au-In_2O_3.

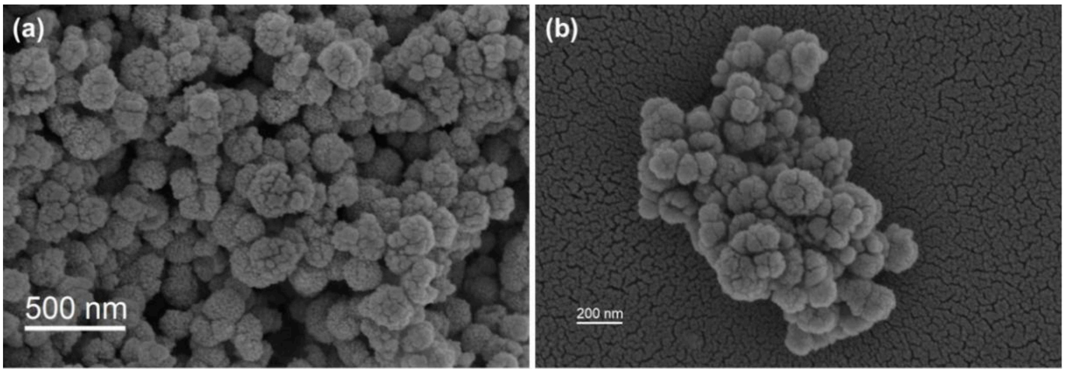

Figure S5. (**a**,**b**) SEM imagesof the pristine In_2O_3 sample.

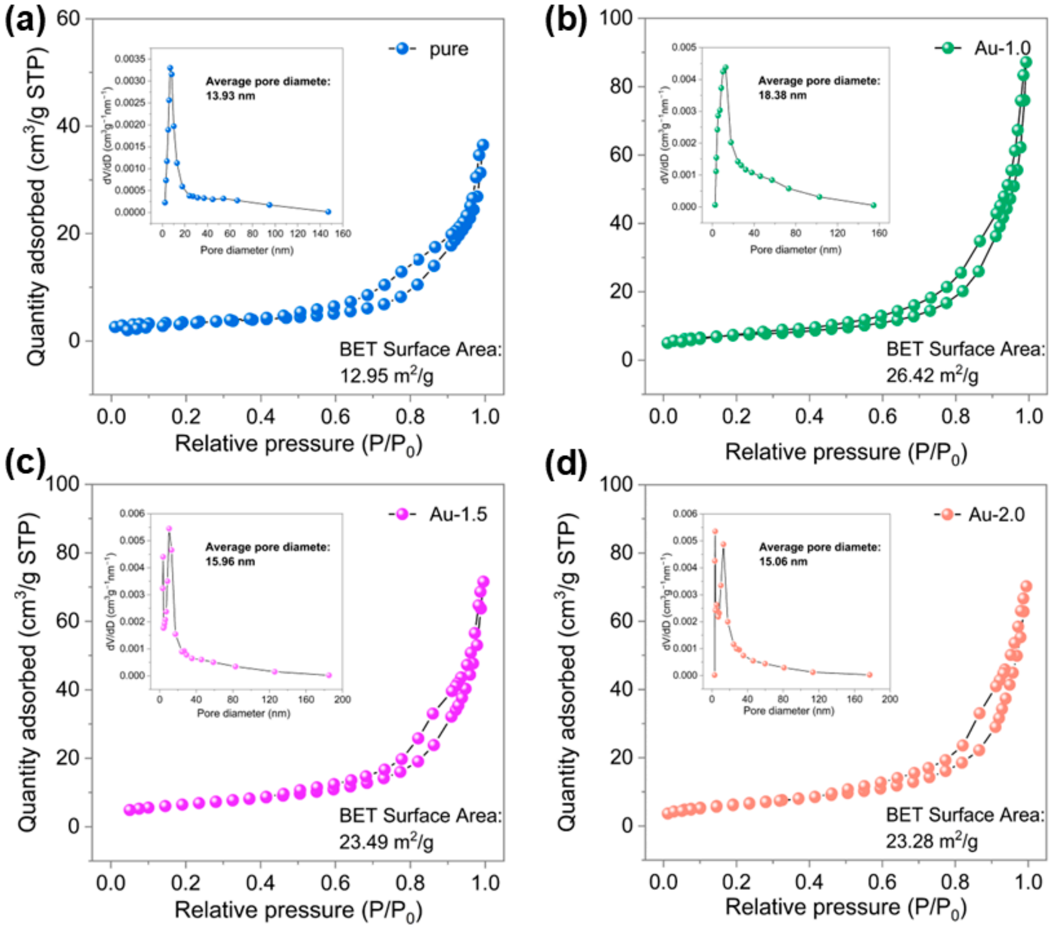

Figure S6. N_2 adsorption desorption isotherms, pore size distributions and BET surface areas of (**a**) pure, (**b**) Au-1.0, (**c**) Au-1.5, (**d**) Au-2.0 respectively.

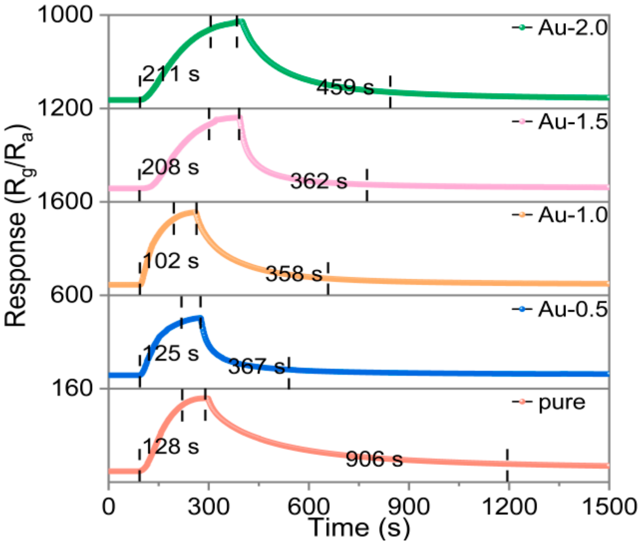

Figure S7. Response and recovery curves toward 1 ppm O_3.

Table S1. Peak position and surface oxygen specie contents of the samples.

Samples	Lattice oxygen (O_L)	Oxygen vacancies (O_V)	Chemisorbed oxygen (O_C)
pure	530.2 eV (55.8%)	531.2 eV (20.8%)	532.2 eV (23.4%)
Au-1.0	530.3 eV (50.1%)	531.4 eV (26.6%)	532.8 eV (23.3%)

Table S2. The comparison in O_3 sensing performance of metal oxide-based sensors between the reported literatures and ourwork

Samples	Concentration	Response	Condition	LOD	Ref.
Mn_3O_4	5 ppm	1.62	Room temperature	200 ppb	[43]
SnO_2	1.3 ppm	1.2	n/a	400 ppb	[44]
$CuAlO_2$	1.15 ppm	≈1.9	250 ºC	200 ppb	[45]
$ZnCo_2O_4$	0.89 ppm	71	200 ºC	80 ppb	[46]
Ag-In_2O_3	1 ppm	≈1.9	25 ºC	100 ppb	[47]
Au-In_2O_3	1 ppm	≈1398.4	30 ºC	100 ppb	This work
